# Supplementary material for: The genome sequence of a pipunculid fly, Nephrocerus scutellatus (Macquart, 1834)
Source: Wellcome Open Res. 2024 Feb 19;9:41. [Version 1] doi: 10.12688/wellcomeopenres.20677.1 (PMC11252644; doi:10.12688/wellcomeopenres.20677.1)
Supplement: Peer review history

# Reviewer report: Reviewer response for version 1

**DOI:** 10.21956/wellcomeopenres.22882.r86774

**Published:** 2024-07-16

**Authors:** Markus Friedrich

**Affiliations:** 1 Wayne State University, Detroit, Michigan, USA

This manuscript reports the successful chromosome-level sequencing of the genome of the pipunculid fly species *Nephrocerus scutellatus* by the Wellcome Sanger Institute. The experimental and computational workflow are described in concise detail. This new high quality genome sequence will be another important contribution to the fields of comparative genomics and biodiversity studies.

 Minor comment: "is" in first sentence of the Background section needs to be de-italicized.

Are sufficient details of methods and materials provided to allow replication by others?

Yes

Is the rationale for creating the dataset(s) clearly described?

Yes

Are the datasets clearly presented in a useable and accessible format?

Yes

Are the protocols appropriate and is the work technically sound?

Yes

Reviewer Expertise:

Comparative genomics

I confirm that I have read this submission and believe that I have an appropriate level of expertise to confirm that it is of an acceptable scientific standard.

# Reviewer report: Reviewer response for version 1

**DOI:** 10.21956/wellcomeopenres.22882.r86769

**Published:** 2024-06-26

**Authors:** Jerome H L Hui

**Affiliations:** 1 The Chinese University of Hong Kong, Hong Kong, Hong Kong

In this data note, Falk and colleagues sequenced and assembled the genome of a dipteran species, commonly known as big-headed fly, *Nephrocerus scutellatus *(Macquart, 1834). This species has been reported in southern England according to Falk and Chandler 2005 (Joint Nature Conservation Committee, ISSN 1473-0154). Molecular data of this species are scarce prior to this report, and are mainly COI sequences deposited to the NCBI database. Therefore, this new genome resource will be useful for further studies, ranging from studying the mechanism of being parasitoids to insect hosts, their population structure, to understanding their evolution with other insects.

 This genome resource is excellent from the summary statistics, with high BUSCO number scores, high sequence continuity (scaffold N50), and majority of sequences contained on the 5 pseudochromosomes (plus sex chromosome and mitochondrion). All in all, this is a valuable contribution.

Are sufficient details of methods and materials provided to allow replication by others?

Yes

Is the rationale for creating the dataset(s) clearly described?

Yes

Are the datasets clearly presented in a useable and accessible format?

Yes

Are the protocols appropriate and is the work technically sound?

Yes

Reviewer Expertise:

Genomics, evolution, invertebrates

I confirm that I have read this submission and believe that I have an appropriate level of expertise to confirm that it is of an acceptable scientific standard.
